# Supplementary material for: Adapting non-invasive human recordings along multiple task-axes shows unfolding of spontaneous and over-trained choice
Source: eLife. 2021 May 11;10:e60988. doi: 10.7554/eLife.60988 (PMC8143794; doi:10.7554/eLife.60988)
Supplement: Supplementary file 1. — The list of task conditions and corresponding regressors of the experiment are shown. The four bold lines are illustrated as examples in Figure 1B. [file elife-60988-supp1.docx]

Supplementary File 1

| Task conditions | | | | Regressors | | | | | | | |
| --- | --- | --- | --- | --- | --- | --- | --- | --- | --- | --- | --- |
| Adaptation stimulus (Right/Left/Red/Green) | Test stimulus | | |  |  |  |  |  |  |  |  |
|  | Context (Motion/  Colour) | Dot motion (Right/  Left) | Dot colour (Red/  Green) | Context (Motion/  Colour) | Switch instruction (Yes/No) | Relevant input (RS) (Yes/No) | Irrelevant input (RS) (Yes/No) | | Response (RS) (Yes/No) | | Choice direction  (Right/  Left) |
| Right | Motion | Right | Red | Motion | No | Yes | No | Yes | | Right | |
| Right | Motion | Right | Green | Motion | No | Yes | No | Yes | | Right | |
| Right | Motion | Left | Red | Motion | No | No | No | No | | Left | |
| Right | Motion | Left | Green | Motion | No | No | No | No | | Left | |
| Right | Colour | Right | Red | Colour | Yes | No | Yes | Yes | | Right | |
| Right | Colour | Right | Green | Colour | Yes | No | Yes | No | | Left | |
| Right | Colour | Left | Red | Colour | Yes | No | No | Yes | | Right | |
| Right | Colour | Left | Green | Colour | Yes | No | No | No | | Left | |
| Left | Motion | Right | Red | Motion | No | No | No | No | | Right | |
| Left | Motion | Right | Green | Motion | No | No | No | No | | Right | |
| Left | Motion | Left | Red | Motion | No | Yes | No | Yes | | Left | |
| Left | Motion | Left | Green | Motion | No | Yes | No | Yes | | Left | |
| Left | Colour | Right | Red | Colour | Yes | No | No | No | | Right | |
| Left | Colour | Right | Green | Colour | Yes | No | No | Yes | | Left | |
| Left | Colour | Left | Red | Colour | Yes | No | Yes | No | | Right | |
| Left | Colour | Left | Green | Colour | Yes | No | Yes | Yes | | Left | |
| Red | Motion | Right | Red | Motion | Yes | No | Yes | Yes | | Right | |
| Red | Motion | Right | Green | Motion | Yes | No | No | Yes | | Right | |
| **Red** | **Motion** | **Left** | **Red** | **Motion** | **Yes** | **No** | **Yes** | **No** | | **Left** | |
| Red | Motion | Left | Green | Motion | Yes | No | No | No | | Left | |
| Red | Colour | Right | Red | Colour | No | Yes | No | Yes | | Right | |
| Red | Colour | Right | Green | Colour | No | No | No | No | | Left | |
| **Red** | **Colour** | **Left** | **Red** | **Colour** | **No** | **Yes** | **No** | **Yes** | | **Right** | |
| Red | Colour | Left | Green | Colour | No | No | No | No | | Left | |
| Green | Motion | Right | Red | Motion | Yes | No | No | No | | Right | |
| Green | Motion | Right | Green | Motion | Yes | No | Yes | No | | Right | |
| **Green** | **Motion** | **Left** | **Red** | **Motion** | **Yes** | **No** | **No** | **Yes** | | **Left** | |
| Green | Motion | Left | Green | Motion | Yes | No | Yes | Yes | | Left | |
| Green | Colour | Right | Red | Colour | No | No | No | No | | Right | |
| Green | Colour | Right | Green | Colour | No | Yes | No | Yes | | Left | |
| **Green** | **Colour** | **Left** | **Red** | **Colour** | **No** | **No** | **No** | **No** | | **Right** | |
| Green | Colour | Left | Green | Colour | No | Yes | No | Yes | | Left | |

**Supplementary Table 1, Task conditions and the corresponding regressors.**

The list of task conditions and corresponding regressors of the experiment are shown. The four bold lines are illustrated as examples in Figure 1B.
